# Editorial: The Role of Glia in Alzheimer's Disease

**DOI:** 10.3389/fneur.2018.01161

**Published:** 2019-01-11

**Authors:** Beatriz G. Pérez-Nievas, Alberto Serrano-Pozo

**Affiliations:** ^1^Department of Basic and Clinical Neuroscience, Institute of Psychiatry, King's College London, London, United Kingdom; ^2^Department of Neurology of the Massachusetts General Hospital, Harvard Medical School, Boston, MA, United States

**Keywords:** Alzheimer's disease, amyloid plaques, astrocytes, chemokines/chemokine receptors, glia, microglia, neurofibrillary tangles (NFTs), neuroinflammation

For decades, Alzheimer's disease (AD) research has focused on the two pathological hallmarks of the disease: amyloid plaques and neurofibrillary tangles. Reactive astrocytes and activated microglia decorating amyloid plaques are other long known pathological features of the AD brain (1–4), yet only recently has the role of glia in AD gained momentum as a research topic (5). This growing interest in glia is primarily fueled by the GWAS discovery of several risk loci in genes related to the innate immune system (6), and by the recent involvement of microglia and astrocytes in synaptic pruning and the modulation of synaptic activity in physiologic conditions (7–10). Indeed, reactive glia has been correlated with both clinical expression and progression of cognitive decline in AD (1, 11). In the 10 articles that form this Frontiers Research Topic, now edited as an eBook, the readers will find an update on some of the most crucial aspects of astrocyte and microglia involvement in AD pathophysiology, as well as some of the most novel and useful tools to study both glial cell types in the context of AD.

We start with a comprehensive review on the role of reactive astrocytes in the disease, highlighting the heterogeneity and complexity of astrocytes in the healthy brain, the molecular signaling pathways involved in astrocyte reaction in AD, the phenotypic changes exhibited by reactive astrocytes in the AD brain, and the consequences of this astrocyte reaction with respect to plaques, tangles, neurons, and synapses (Perez-Nievas and Serrano-Pozo). Next, Garcia-Esparcia et al. compare the astrocyte reaction present in AD and dementia with Lewy bodies (DLB) brains by quantifying both protein and mRNA levels of several astrocyte markers such as glial fibrillary acidic protein (GFAP), excitatory amino acid transporter 2 (EAAT2/GLT-1), and aldehyde dehydrogenase 1 L1 (ALDH1L1). They observed a non-significant reduction of EAAT2/GLT-1 protein levels and a normal EAAT2/GLT-1 immunoreactivity around plaques, suggesting limited consequences of astrocyte reaction for glutamate transport in AD.

The Frontiers Research Topic/eBook switches then gears to focus on the role of microglia in AD. Navarro et al. summarize their recent findings comparing microglia from the hippocampus of APP-overexpressing transgenic mice and human AD brains (12). They postulate that, while microglia becomes uniformly activated and pro-inflammatory in the hippocampus of mouse models of amyloid plaque deposition, a subset of microglia from the human AD hippocampus might be dysfunctional and exhibit an attenuated inflammatory response, and even degenerate due to the toxicity mediated by soluble tau oligomers. Zhou et al. review the physiology of triggering receptor expressed on myeloid cell 2 (TREM2) and its implication in amyloid plaque and tangle formation from studies on *Trem2* deficient AD mouse models. They also review their recent finding that TREM2 enhances microglial metabolism through the mammalian target of rapamycin (mTOR) pathway (13), suggesting that the AD-linked *TREM2* variants (14, 15) can modulate AD pathogenesis through an aberrant microglial metabolism. Guedes et al. review the contributions of microglial and monocyte chemokines and their receptors (CCL2/CCR2, CX3CL1/CX3CR1, CCL5/CCR5, CXCL10/CXCR3, and CXCL1/CXCR2) to amyloid and tau pathologies. Thei et al. contribute with a review of the ion channels expressed by homeostatic microglia, their potential disruption in activated microglia in AD, and how human inducible pluripotent stem cell (hiPSC)-derived microglia could be a better tool than primary microglial cultures to elucidate the role of these ion channels. And, finally, Chun et al. summarize their experience with novel *in vitro* approaches to study glia, including microfluidic devices with human microglia exposed to Aβ to investigate microglial chemotaxis (16), and a 3D organotypic AD brain model (17) consisting of culturing neurons, microglia and astrocytes from immortalized human AD neural progenitor cells or hiPSC-derived neural progenitor cells in a 3D microfluidic platform.

Lastly, the Frontiers Research Topic/eBook deals with the imaging methods available to study reactive glia. Edison et al. review the literature on PET imaging of reactive glia in both human AD subjects and AD mouse models. PET radiotracers targeting the translocator protein of 18 KDa (TSPO) have been widely used for almost two decades to image activated microglia *in vivo* (18), whereas [^11^C]deuterium-L-deprenyl ([^11^C]DED)—an irreversible inhibitor of monoamine oxidase B—has recently been introduced to image reactive astrocytes (19), which are known to up-regulate this enzyme (20). Hierro-Bujalance et al. provide an update on the methodology of intravital multiphoton microscopy and its applications to image microglia *in vivo* in AD mouse models. Examples of key observations using this technique include the microglia chemotaxis toward amyloid plaques after these are formed, its limited role in controlling plaque growth, and its activation and participation in plaque clearance upon treatment with anti-Aβ antibodies. Kelly et al. address the current applications of intravital multiphoton microscopy to image astrocytes *in vivo* in AD mouse models. These include, among others, the topical use of the dye sulforhodamine-101 (SR-101) to study astrocyte morphology and distribution (21), and the virally-mediated expression of genetically-encoded calcium indicators to track astrocyte calcium dynamics as a functional read-out (e.g., calcium waves at both intracellular and network scales) (22). Practical examples of these functional studies include the investigation of spontaneous calcium transients as a function of proximity to amyloid plaques and cerebral amyloid angiopathy (CAA)-affected vessels, and the examination of evoked calcium transients in paradigms of functional hyperemia.

In summary, although acknowledging that the topic of glial cells in AD is a rapidly evolving field, we believe that the present Frontiers Research Topic/eBook will provide the interested readers with the most recent developments on the role of reactive astrocytes and activated microglia to AD pathophysiology, and the latest technical advances to study and image these glial cells *in vitro* and *in vivo* in AD patients and mouse models.

## Author Contributions

All authors listed have made a substantial, direct and intellectual contribution to the work, and approved it for publication.

### Conflict of Interest Statement

The authors declare that the research was conducted in the absence of any commercial or financial relationships that could be construed as a potential conflict of interest.
